# Impact of vaccination with different types of rotavirus vaccines on the incidence of intussusception: a randomized controlled meta-analysis

**DOI:** 10.3389/fped.2023.1239423

**Published:** 2023-07-31

**Authors:** Guoyong Wang, Kaijun Zhang, Rensen Zhang, Xiangru Kong, Chunbao Guo

**Affiliations:** ^1^ Department of Pediatrics, Women and Children's Hospital of Chongqing Medical University, Chongqing, China; ^2^ Department of Pediatric General Surgery, Children's Hospital, Chongqing Medical University, Chongqing, China; ^3^ National Clinical Research Center for Child Health and Disorders, China International Science and Technology Cooperation Base of Child Development and Critical Disorders, Chongqing Key Laboratory of Pediatrics, Chongqing Medical University, Chongqing, China; ^4^ Department of Pediatrics, Chongqing Health Center for Women and Children, Chongqing, China

**Keywords:** rotavirus vaccine, intussusception, incidence, RCT, randomized clincial trial

## Abstract

**Background:**

Intussusception is a prevalent pediatric issue causing acute abdominal pain, with potential links to rotavirus vaccines. The variety of these vaccines has grown in recent years. This meta-analysis study aims to evaluate the impact of various rotavirus vaccines on intussusception incidence.

**Methods:**

We executed a thorough search across databases like PubMed, Cochrane Library, Embase, and Web of Science, leading to the selection of 15 credible randomized controlled trials (RCTs) that encompass various types of rotavirus vaccines. From each study, we extracted essential details such as vaccine types and intussusception occurrences. We assessed the risk of bias using the Cochrane Collaboration's tool, conducted statistical analysis with R (version 4.2.3), determined relative risk (RR) using a random effects model, and performed a subgroup analysis for vaccines of differing brands and types.

**Results:**

We included 15 randomized controlled studies from various countries. While intussusception incidence differed between vaccinated and control groups, this difference was not statistically significant. The overall risk ratio (RR), calculated using a random effects model, was 0.81, with a 95% confidence interval of [0.53, 1.23]. This crossing 1 shows that vaccination didn't notably change disease risk. Additionally, the 0% group heterogeneity suggests consistency across studies, strengthening our conclusions. Subgroup analysis for different vaccine brands and types (RV1 (Rotarix, Rotavac, RV3-BB), RV3 (LLR3), RV5 (RotasiiL, RotaTeq), and RV6) showed no significant variation in intussusception incidence. Despite variations in RR among subgroups, these differences were not statistically significant (*P* > 0.05).

**Conclusions:**

Our study indicates that rotavirus vaccination does not significantly increase the incidence of intussusception. Despite varying impacts across different vaccine brands and types, these variations are insignificant. Given the substantial benefits outweighing the risks, promoting the use of newly developed rotavirus vaccines remains highly valuable.

**Systematic Review Registration:**

www.crd.york.ac.uk/prospero/, Identifier CRD42023425279.

## Introduction

Intussusception refers to a section of the intestine telescoping into the lumen of the adjacent distal intestine, causing intestinal obstruction. Intussusception represents one of the most prevalent acute abdominal conditions among infants and toddlers, and it also stands as a primary cause for emergency surgical intervention in the field of pediatric surgery (1). The incidence of intussusception exhibits regional and population variations, typically ranging from 25 to 100 cases per 100,000 children (2). Intussusception mostly occurs in infants aged 3–12 months, more common in males than females, with the ileocecal part being the main site (3). Intussusception presents with various clinical manifestations, predominantly characterized by paroxysmal abdominal pain, vomiting, bloody stools, and currant jelly-like stools, as well as the presence of abdominal masses (4). The diagnosis of intussusception mainly relies on clinical manifestations and imaging examinations, among which ultrasound examination is the first-choice method with high sensitivity and specificity (5). Intussusception is primarily managed through non-surgical and surgical treatments. Non-surgical treatment includes gas or liquid enema reduction, suitable for early intussusception children without complications such as perforation, necrosis, and infection (6). Surgical treatment includes laparotomy, intestinal reduction or resection of necrotic intestinal segments, suitable for children with failed non-surgical treatment or contraindications (7).

Rotavirus is a prominent pathogen responsible for acute gastroenteritis in infants and young children, contributing to approximately 215,000 deaths annually among children under the age of 5 worldwide (8). Rotavirus infection can cause clinical manifestations such as diarrhea, vomiting, fever, etc., severe cases can lead to dehydration, acidosis, electrolyte disorders and other complications, The prevention of rotavirus infection mainly depends on the immunization of live rotavirus vaccine, the vaccination of rotavirus (RV) vaccine significantly mitigates the incidence and severity of rotavirus-related gastroenteritis, thereby diminishing mortality rates (9). There are two kinds of live rotavirus vaccines commonly used in the market: RV1 (Rotarix) and RV5 (Rotateq), made from human-derived and human-bovine recombined rotavirus strains, respectively. The World Health Organization has recommended these two live rotavirus vaccines as a widely used component of routine immunization programs globally, while demonstrating good immunogenicity and safety (10, 11). In recent years, as the number and variety of rotavirus vaccine brands have grown, the potential link between rotavirus vaccine administration and the incidence of intussusception has become a focal point in public health debates (12, 13).

Particularly following the introduction of the rotavirus vaccine into the market, certain market surveillance studies have identified an increased risk of intussusception within 7 days after vaccination in some infants and young children (14). This discovery has generated substantial attention, given that the first-generation oral tetravalent rotavirus vaccine (RRV-TV) was recalled from the market in 1999 due to its link with intussusception (15). As the assortment of rotavirus vaccine brands expands on the market, numerous cohort and case-control epidemiological studies have appraised the correlation between the rotavirus vaccine and intussusception. However, their findings have demonstrated inconsistency (16, 17). In addition, the impact of different brands and types of oral rotavirus on the increased risk of intussusception is not yet clear.

Thus, this paper seeks to examine the influence of rotavirus vaccine immunization on the frequency of intussusception via a systematic evaluation and meta-analysis of the existing literature, particularly focusing on the effects of varying brands and types of rotavirus vaccines.

## Methods

### Data sources and search strategy

We will search the following databases for relevant studies: PubMed, Embase, Cochrane Library, Web of Science. We will use the following keywords for the search: (“rotavirus vaccine” OR “RV1” OR “RV5” OR “Rotarix” OR “Rotateq”) AND (“intussusception” OR “invagination”) AND “randomized controlled trial”. The search will be restricted from 2000 to 2023, with English language restriction. We will also refer to the references of published studies to find other studies that may meet the inclusion criteria. This study adheres to the PRISMA guidelines and has been pre-registered in PROSPERO under the registration number CRD42023425279. The registration provides a comprehensive overview of our research objectives and procedures (18).

### Inclusion and exclusion criteria

We will incorporate studies that fulfill the following criteria: (1) Randomized controlled trial design; (2) The study subjects are children who have been vaccinated with oral rotavirus vaccine; (3) The study outcomes include the incidence (per person) or relative risk of intussusception; (4) Provides sufficient data for meta-analysis.

We will exclude: (1) Repeatedly published or secondary published articles, systematic reviews or meta-analyses; (2) Case reports or observational studies; (3) And literature that is irrelevant to the topic of this article or of poor quality.

### Data extraction

We will extract the following data from each study that meets the inclusion criteria: (1) Basic information, including the first author, year of publication, research design, research location, etc.; (2) Baseline characteristics of participants, such as age, gender, brand and type of vaccine received, and dose vaccination time, the incidence of intussusception (per person), the success rate of reduction, and surgery rate, etc.; (3) The incidence and relative risk or odds ratio of intussusception, as well as the related confidence intervals and *P* values. Data extraction will be carried out independently by two researchers, GW and KZ. In case of any discrepancies, a third researcher, CG or XK, will make the final decision.

### Quality assessment

The quality of the included studies was evaluated using the Cochrane Collaboration's risk of bias tool. This tool assesses potential biases in seven domains: random sequence generation (selection bias), allocation concealment (selection bias), blinding of participants and personnel (performance bias), blinding of outcome assessment (detection bias), incomplete outcome data (attrition bias), selective reporting (reporting bias), and other potential biases. For each domain, the studies were rated as “low risk”, “unclear”, or “high risk”.

The assessment was conducted independently by two researchers, GW and RZ. In case of disagreement, a third researcher, CG or XK, was consulted to make a final decision (19).

## Results

### Search results

**Figure 1:**
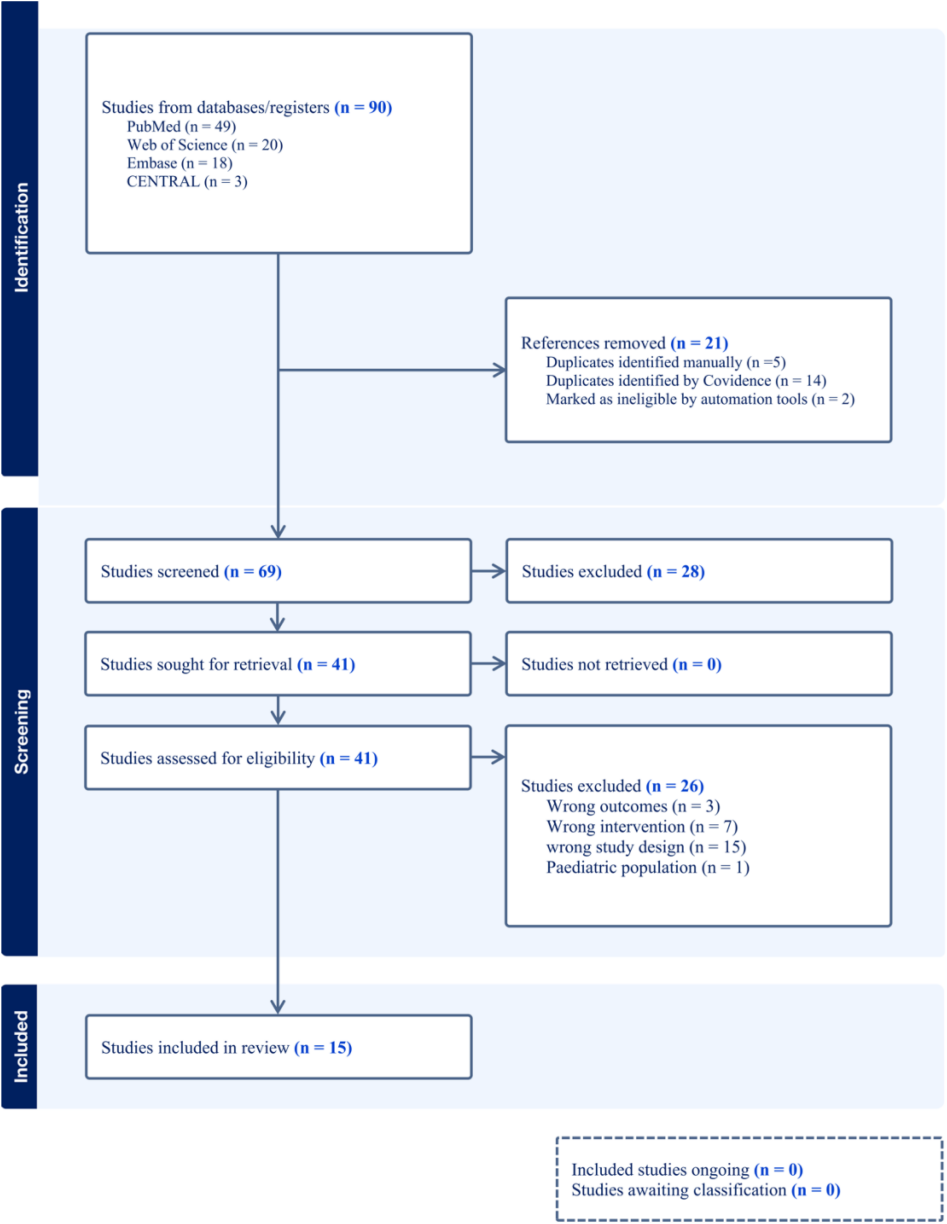
Flowchart of search strategy.

Our search initially identified a total of 90 relevant studies, which included 49 from PubMed, 18 from Embase, 20 from Web of Science, and 3 from the Cochrane Library. We screened these studies by their titles and abstracts and excluded 21 duplicates. After reviewing the full texts of the remaining 69 studies, we excluded 54 that did not meet our inclusion criteria. These excluded studies consisted of 3 with incorrect outcomes, 7 with inappropriate interventions, and 15 with unsuitable study designs, such as non-randomized controlled trials. Moreover, we excluded one study that focused on a pediatric population due to its limited sample size, which could not provide sufficient statistical power to support the reliability of its findings. Therefore, our final analysis incorporated 15 high-quality randomized controlled trials (Figure 1).

### Study characteristics and quality assessment

Among the 15 included papers, all were randomized controlled trials conducted in multiple countries. These countries encompassed the United States, Mexico, Brazil, Australia, New Zealand, Vietnam, India, China, Finland, and 11 Latin American countries, namely Argentina, Brazil, Chile, Colombia, Dominican Republic, Honduras, Mexico, Nicaragua, Panama, Peru, and Venezuela. Among them, 8 papers provided data on the incidence, relative risk, or odds ratio of intussusception following RV1 vaccination, while 5 papers reported the incidence, relative risk, or odds ratio of intussusception after RV5 vaccination. and 2 papers respectively reported the incidence of intussusception in people vaccinated with RV3 (LLR3) and R V6 rotavirus vaccines. The 15 studies included covered a total of 99,194 children vaccinated with rotavirus vaccines (Table 1).

**Table 1:** Characteristics of the included randomized clinical trials.

Rct_Id	Source	Countries or Regions	Vaccine	Study period	Registration No.	Queue, No.	Vaccination schedule	Vaccine Group	Placebo Group
#6	Bhandari. et al. (20)	Delhi, Pune and Vellore in India	RV1 (Rotavac)	March 2011—September 2013	NCT01305109	1	6–14 wk	4,532	2,267
#7	Bines et al. (21)	Indonesia	RV1 (RV3-BB)	January 2013-July 2016	ACTRN12612001282875	1	0–5 d and 8–10 wk.	1,091	549
#10	Chang et al. (22)	Taiwan	RV5 (RotaTeq)	April 2003—June 2004	NA	1	6–12 wk	95	93
#11	Chilengi et al. (23)	Zambia	RV1 (Rotavac)/RV1 (Rotatvc 5D) RV1 (Rotarix)	January 2019—October 2019	NCT03602053	2	6–14 wk and 6–10 wk	150/150/150	0
#13	Christie et al. (24)	Jamaica	RV5 (RotaTeq)	February 2002—October 2005	NA	1	2–6 mo	904	898
#16	Coldiron et al. (25)	Niger	RV5 (RotasiiL)	2016–2018	NCT02145000	1	6–14 wk	2,042	2,044
#33	Linares et al. (26)	Multiple countries in Latin America	RV1 (Rotarix)	August 2003—October 2005	NCT00140673	1	2–4 mo	7,669	7,514
#35	Middleton et al. (27)	Australia	RV1 (Rotarix)	March 2018—August 2020	NCT02941107	1	NA	128	125
#36	Mo et al. (28)	China	RV5 (RotaTeq)	May 2014—October 2014	NCT02062385	1	6–12 wk	2015	2019
#38	Puha et al. (29)	Asia (Hong Kong, Singapore, Taiwan)	RV1 (Rotarix)	December 2003—August 2005	NCT00197210	1	6–12 wk	5,259	5,249
#39	Ruiz-Palacios et al. (30)	11 Latin American countries, Finland	RV1 (Rotarix)	August 2003-March 2004	NCT00139347 and NCT00263666	1	2–4 mo	31,673	31,552
#44	Thiam et al. (31)	Vietnam	RV1 (Rotavin)/Rv1 (Rotavin-M1)	March 2019—January 2020	NCT03703336	1	NA	551/274	0
#48	Vesikari et al. (32)	11 Countries	RV5 (RotaTeq)	2001–2004	NCT00090233	1	6–12 wk	34,644	34,630
#49	Wu et al. (33)	China	RV6	2019–2021	NA	1	6–12 wk	3,198	3,193
#50	Xia et al. (34)	China	RV3 (LLR3)	2012–2014	NCT01738074	1	6–14 wk	4,993	4,992

Rct_id represents the sequence number of each literature reference, generated by Review Manager 5.4 software.

The quality assessment of the included studies was conducted using the Cochrane Collaboration's risk of bias tool, revealing most low-risk ratings across all assessed domains. These low-risk ratings highlight the high quality of the randomized control trials included in our meta-analysis, which are characterized by strong random sequence generation, good allocation concealment, adequate blinding of participants and personnel, appropriate blinding of outcome assessment, complete outcome data, and non-selective reporting.

**Figure 2:**
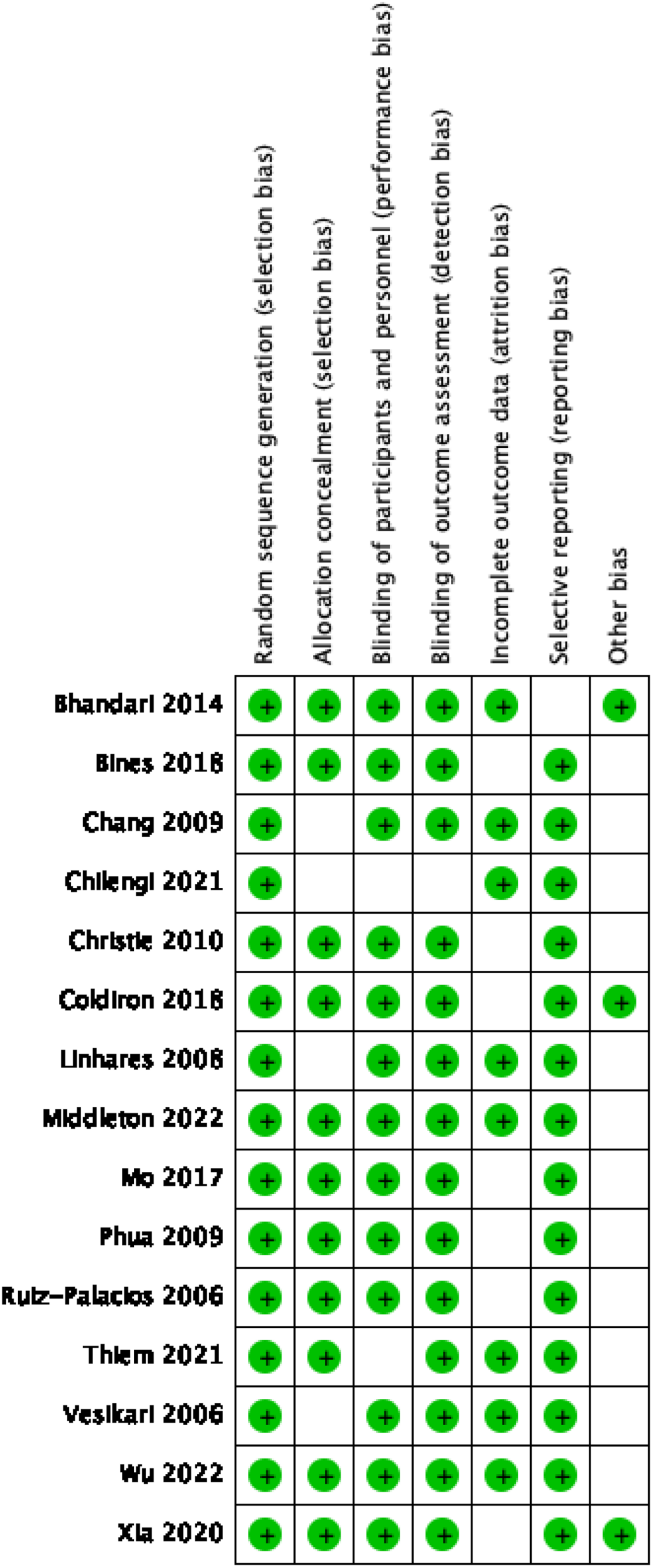
Risk of bias summary.

However, a minority of studies, particularly those with smaller sample sizes, received “unclear” risk ratings in some categories. These ratings indicate that the details provided in these studies were not sufficient to confidently assess the risk of bias in these domains. The specific risk of bias assessment results for each study are displayed in Figure 2.

Thus, while our meta-analysis is largely based on high-quality studies, the potential bias in these smaller studies should be considered when interpreting our results.

### Main results

**Figure 3:**
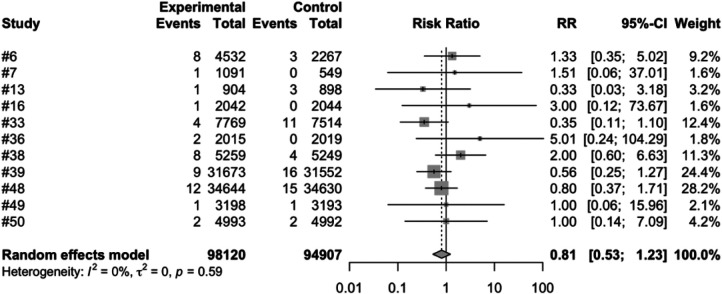
Random effects model meta-analysis of rotavirus vaccine and intussusception randomized controlled trials.

**Figure 4:**
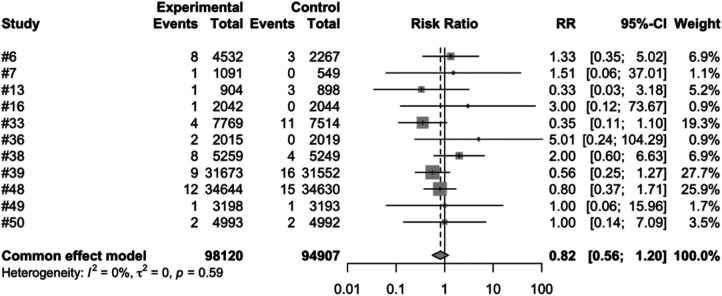
Common effect model meta-analysis of rotavirus vaccine and intussusception randomized controlled trials.

We conducted a meta-analysis using data from the included articles, which encompassed a total of 15 studies involving 194,893 participants. Among these participants, 99,194 were allocated to the vaccine group, while 95,699 were assigned to the control group. After excluding the confounding items where both the intervention group and the control group had zero events, we used a random effects model to perform a combined analysis of the studies. The results showed (Table 2) that the vaccine group had some influence on the incidence of intussusception, but the influence did not reach a significant level statistically. In the random effects model, the overall risk ratio (RR) was 0.81, with a 95% confidence interval of [0.53, 1.23], suggesting that the vaccination did not significantly alter the risk of intussusception (Figure 3). Similarly, in the fixed-effects model, the risk ratio also showed similar results (Figure 4).

**Table 2:** Meta-analysis results of the risk of intussusception after rotavirus vaccination.

RCT_ID	Vaccine	Control	Events intervention	Events control	N_Intervention	N_Control	RR	CI (95%)
#6	RV1 (Rotavac)	Placebo	8	3	4,532	2,267	1.33	[0.35,5.03]
#7	RV1 (RV3-BB)	Placebo	1	0	1,091	549	lnf	[NaN,Inf]
#10	RV5 (RotaTeq)	Placebo	0	0	95	93	NaN	NaN
#11	RV1 (Rotavac)	RV1 (Rotarix)	0	0	150	150	NaN	NaN
#11	RV1 (Rotatvc 5D)	RV1 (Rotarix)	0	0	150	150	NaN	NaN
#13	RV5 (RotaTeq)	Placebo	1	3	904	898	0.33	[0.03,3.18]
#16	RV5 (RotasiiL)	Placebo	1	0	2,042	2,044	lnf	[NaN,Inf]
#33	RV1 (Rotarix)	Placebo	4	11	7,769	7,514	0.35	[0.11,1.10]
#35	RV1 (Rotarix)	Placebo	0	0	128	125	NaN	NaN
#36	RV5 (RotaTeq)	Placebo	2	0	2,015	2,019	lnf	[NaN,Inf]
#38	RV1 (Rotarix)	Placebo	8	4	5,259	5,249	2	[0.60,6.63]
#39	RV1 (Rotarix)	Placebo	9	16	31,673	31,552	0.56	[0.25,1.27]
#44	Rv1 (Rotavin)	RV1 (Rotavin-M1)	0	0	551	274	NaN	NaN
#48	RV5 (RotaTeq)	Placebo	12	15	34,644	34,630	0.8	[0.37,1.71]
#49	RV6	Placebo	1	1	3,198	3,193	1	[0.06,15.96]
#50	RV3 (LLR3)	Placebo	2	2	4,993	4,992	1	[0.14,7.10]

Risk and 95% confidence intervals were calculated using the Mantel–Haenszel method, with a random effects model used to pool data. Randomized clinical trials with no cases of intussusception in both the vaccine and placebo groups were not included in the relative risk statistics but were included in the risk difference statistics.

“Placebo” refers to a substance that, while not containing any rotavirus vaccine, has the same volume as the vaccine and is administered in an identical dosage form and method.

“Events Intervention” refers to the number of instances of intussusception occurring in children who received the rotavirus vaccine, while “Events Control” denotes the number of intussusception incidents in children who were given a placebo. “N_Intervention” represents the total number of children who were vaccinated with the rotavirus vaccine, and “N_Control” signifies the total number of children who received the placebo.

### Subgroup analysis

In our study examining the effect of rotavirus vaccination on the occurrence of intussusception, we conducted subgroup analyses on various vaccine brands and major vaccine types.

**Figure 5:**
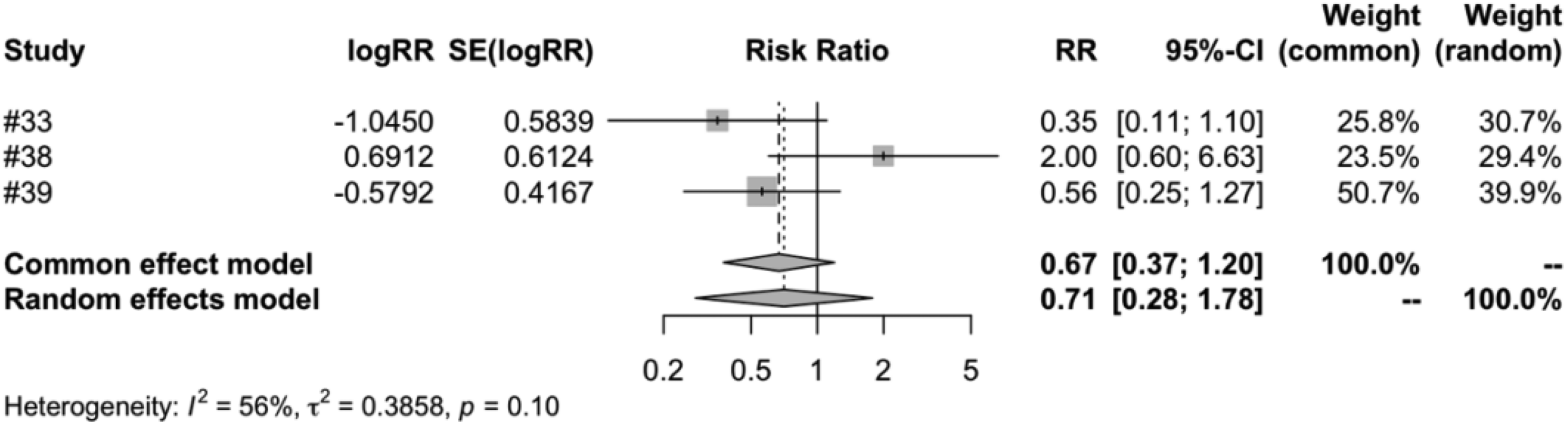
RV1 (Rotarix) subgroup meta-analysis of rotavirus vaccine and intussusception randomized controlled trials.

**Figure 6:**
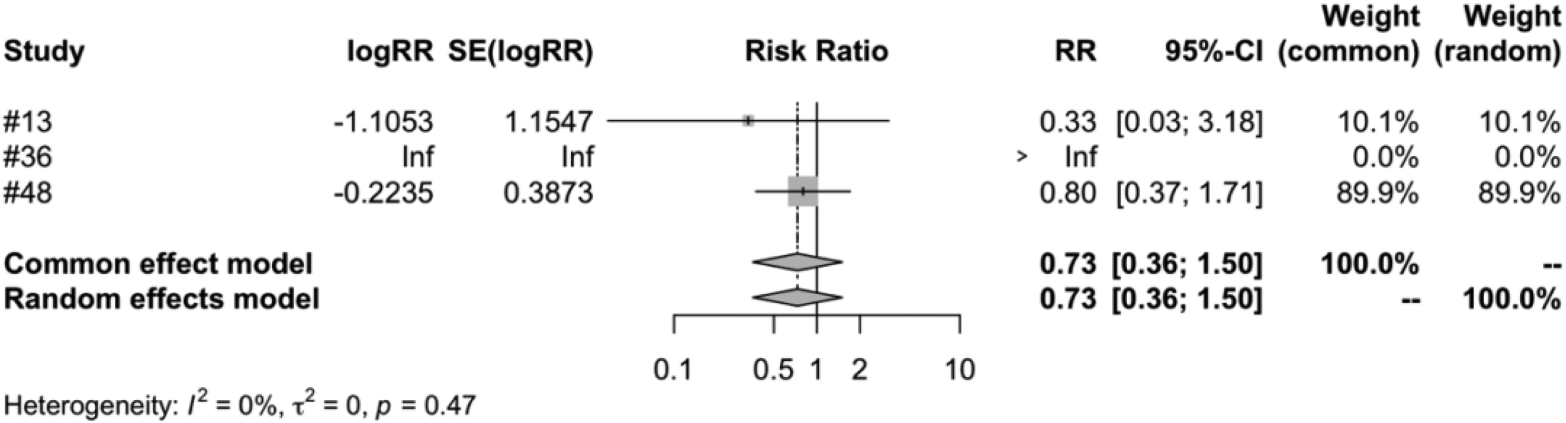
RV5 (RotaTeq) subgroup meta-analysis of rotavirus vaccine and intussusception randomized controlled trials.

In the subgroup analysis of different vaccine brands, we examined 7 types of vaccines: RV1 (Rotarix), RV1 (Rotavac), RV1 (RV3-BB), RV3 (LLR3), RV5 (RotasiiL), RV5 (RotaTeq) and RV6. The meta-analysis of three studies on RV1 (Rotarix) revealed a risk ratio (RR) of intussusception in the vaccinated group compared to the control group to be 0.7051, accompanied by a 95% confidence interval ranging from 0.2793 to 1.7803. Despite an *I*^2^ heterogeneity of 56.3%, and a *P* value of 0.4596, the result did not reach statistical significance (Figure 5). RV1 (Rotavac) included only one study, with a risk ratio of 1.3339, a 95% confidence interval of [0.3539, 5.0280], and a *P* value of 0.6704, but the result was also not significant. For RV1 (RV3-BB) and RV5 (RotasiiL), It was impossible to calculate the risk ratio as the control group did not report any instances of intussusception. Both RV3 (LLR3) and RV6 included one study each, and their findings suggest that the vaccination did not significantly influence the incidence of intussusception. The meta-analysis of three studies focusing on RV5 (RotaTeq) demonstrated a risk ratio (RR) of intussusception in the vaccinated group vs. the control group of 0.7315. This was within a 95% confidence interval between 0.3562 and 1.5023. Despite a *P* value of 0.3944 and an *I*^2^ variability of 0.0%, the findings did not yield statistical significance (Figure 6).

**Figure 7:**
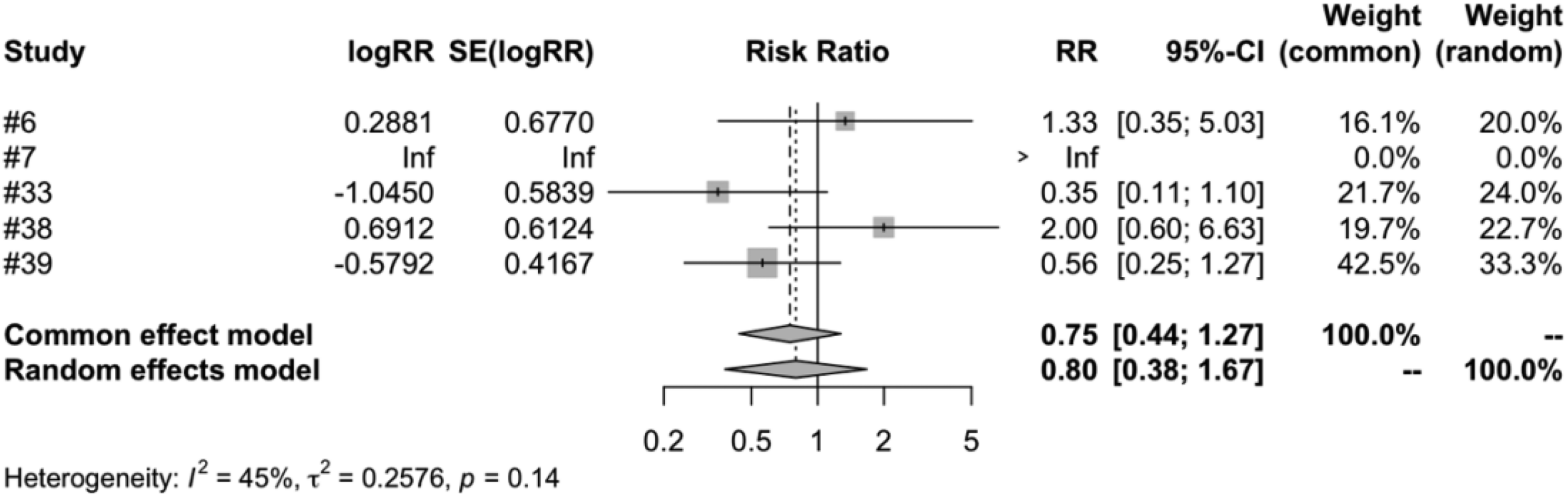
RV1 subgroup meta-analysis of rotavirus vaccine and intussusception randomized controlled trials.

**Figure 8:**
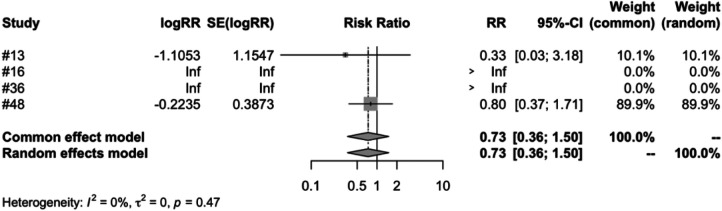
RV5 subgroup meta-analysis of rotavirus vaccine and intussusception randomized controlled trials.

In the subgroup analysis of different vaccine types, we examined four types of vaccines: RV1, RV3, RV5 and RV6. The meta-analysis of five studies on RV1 indicated that the vaccinated group's risk ratio (RR) of intussusception, compared to the control group, was 0.7955. This figure was backed by a 95% confidence interval ranging from 0.3786 to 1.6716. Despite the *P*-value measuring at 0.5459 and an *I*^2^ variability of 45.0%, the results were not statistically significant (Figure 7). Both RV3 and RV6 were each covered in a single study, and the results suggest that the vaccination did not have a significant impact on the incidence of intussusception. The meta-analysis of four studies on RV5 showed that the risk ratio (RR) of intussusception in those vaccinated, relative to the unvaccinated, was 0. 7315. The result had a 95% confidence interval ranging from 0.3562 to 1.5023. Although the *P*-value was 0.3944 and an *I*^2^ heterogeneity of 0.0%, the findings were not statistically significant (Figure 8).

### Heterogeneity analysis

We used the tau^2^, tau, *I*^2^ and H statistics to quantify the heterogeneity of the studies. The results showed that tau^2^ was 0, tau was 0, *I*^2^ was 0.0%, and H was 1.00. These results indicate that the heterogeneity between studies is very small. We tested for heterogeneity, with a Q value of 8.88, degrees of freedom of 11, and a *P* value of 0.6327. This indicates that the differences between studies are not statistically significant.

### Sensitivity analysis and bias risk assessment

A comprehensive sensitivity analysis was conducted to assess the robustness and reliability of the meta-analysis results. This analysis involved two key steps:

We systematically excluded each study in sequence to evaluate their influence on the overall findings of the meta-analysis. The analysis was executed using the Mantel–Haenszel method and the restricted maximum likelihood estimation of tau^2^. The results demonstrated minimal alteration in the relative risk (RR) and 95% confidence interval (CI) when individual studies were sequentially excluded from the analysis, indicating the stability of the results across the included studies.

Furthermore, we incorporated the extreme values of the 95% confidence intervals for the intussusception incidence risk ratio into our sensitivity analysis. This allowed us to assess the possible impact of these extreme scenarios on our conclusions. The findings from this analysis reinforced the robustness of our results, even under these extreme conditions.

The results of these comprehensive sensitivity analyses suggest that our meta-analysis findings are robust and not significantly influenced by individual studies or extreme estimates, thereby indicating the overall stability and reliability of the results.

**Figure 9:**
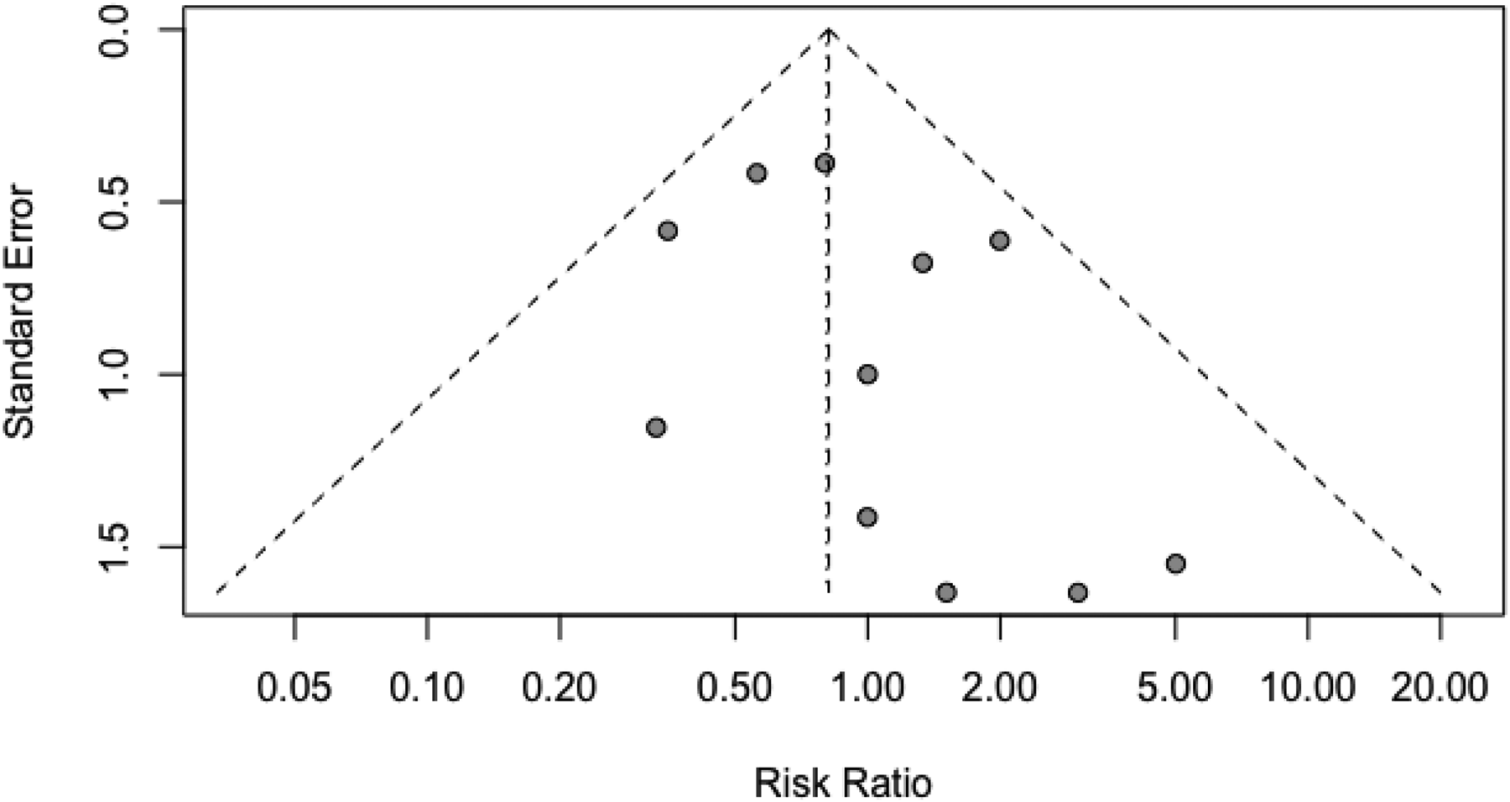
The funnel plot displays the distribution of randomized controlled trials.

We utilized the Egger regression test and funnel plot to evaluate the risk of publication bias in the included literature. The analysis revealed that all the *P*-values were greater than 0.05, and the funnel plot exhibited a symmetrical distribution. These findings indicate the absence of significant publication bias (Figure 9).

## Discussion

Intussusception, a potentially fatal pediatric condition, has been suggested to be associated with the Rotavirus (RV) vaccine based on recent findings. Due to this possible side effect, vigilant monitoring of intussusception progression is recommended post-RV vaccination. Our systematic review and meta-analysis of randomized controlled trials found no considerable correlation between the incidence of intussusception and RV vaccination. The meta-analysis included randomized controlled trials using RV1 (Rotarix), RV1 (Rotavac), RV1 (RV3-BB), RV1 (Rotavin-M1), RV1 (Rotavin), RV3 (LLR3), RV5 (Rotasiil), RV5 (RotaTeq), and RV6. Subgroup analysis of data for various vaccine brands and types, as well as risk estimates for intussusception occurrence after vaccination, found no association between RV vaccine administration and the risk of intussusception, consistent with the results of some previous studies (35–37).

In our research on RV vaccine brands, we included six globally marketed RV vaccines (oral attenuated live vaccines), four of which are pre-certified by WHO, including:RotaTeq (38) (RV5, manufactured by Merck, pre-certified in 2008), Rotarix (39) (RV1, produced by GlaxoSmithKline, pre-certified in 2009), Rotavac (40) (RV1, created by Bharat Biotech, pre-certified in 2018) and Rotasiil (41) (RV5, formulated by the Serum Institute of India, pre-certified in 2018). The most recent vaccines to receive WHO pre-certification, namely Rotavac and Rotasiil, are currently only deployed in India and Palestine. Also, the RV3-BB (21) (RV1) vaccine developed in Australia and three other vaccines are only used in their producing countries: RV1 (Rotavin-M1) (42) in Vietnam, RV3 (LLR3) (34) and RV6 (33) in China. This meta-analysis incorporated all the mentioned vaccines, with subgroup analysis conducted to investigate the effect of different types of RV vaccines on the incidence of intussusception. Nevertheless, RV1 (Rotavin-M1) from Vietnam, which was evaluated against RV1 (Rotavin) in a control study lacking a blank control, was excluded from further analysis due to the limited data (*n* = 825) and the absence of intussusception cases. In addition, we included China's latest hexavalent RV vaccine RV6 in our study. Our subgroup analysis found that there was no significant relationship between the administration of different brands of RV vaccines and the occurrence of intussusception, which may be related to the fact that many vaccines, RV3 (LLR3), RV6, RV (Rotavin-M1) only had a single randomized controlled trial. We hope to see more randomized controlled studies on these vaccine inoculations in the future.

In performing subgroup analyses on various types of rotavirus vaccines, we evaluated the risk of intussusception following the administration of four specific rotavirus vaccines: RV1, RV3, RV5, and RV6. Among these, RV1 includes five randomized controlled studies. RV1 comprises RV1 (Rotarix), RV1 (Rotavac), and RV1 (RV3-BB). RV1 (Rotarix) contains the G1P (8) strain, RV1 (Rotavac) vaccine is derived from naturally attenuated human RV G9P (11) strain, and RV1 (RV3-BB) is a rotavirus vaccine developed from a human neonatal RV strain G3P (6), which was isolated from the feces of asymptomatic infected infants. There is also RV1 (Rotavin-M1) from Vietnam, which is also derived from the G1P (8) strain. RV3 includes one study RV3 (LLR3) that is based on the previously marketed single-price lamb P (12) G10 RV vaccine. Through gene recombination technology, the G2, G3, and G4 human RVs are recombined with LLR to obtain three human-lamb RV recombinant strains each containing the VP7 gene of G2, G3, and G4 human RVs, covering several common RV serotypes (G2, G3, G4). RV5 includes RV5 (RotasiiL) and RV5 (RotaTeq). The RV5 (Rotasiil) vaccine, a lyophilized pentavalent RV vaccine, is produced by the Serum Institute of India. This vaccine originates from naturally attenuated human-bovine RV strains G1, G2, G3, G4, and G9 cultivated in Vero cells. The RotaTeq vaccine, created by Merck & Co., is a five-component bovine-human RV recombinant vaccine. It was recommended for use in children worldwide by the WHO in 2009 and contains five human-bovine RV recombinant strains: G1, G2, G3, G4, and P1A (8). RV6 hexavalent bovine-human recombinant RV attenuated live vaccine contains six popular strains serum: G1, G2, G3, G4, G8, and G9. There is no correlation between the occurrence of intussusception and different types of rotavirus vaccines. This finding is consistent with results reported in previous literature. Although variations exist in the incidence of intussusception among different types of rotavirus vaccines, these differences are not significant (17, 30).

Our findings, demonstrating no significant correlation between the incidence of intussusception and rotavirus vaccination irrespective of variables such as vaccine brand and type, offer robust evidence to the safety assessment of rotavirus vaccine immunization (43). While we didn't directly examine the preventive benefits of the vaccination, namely averting severe complications like diarrhea, dehydration, and fatalities due to rotavirus infection, these benefits have been consistently highlighted in previous research. It's crucial to underscore that such benefits substantially counterbalance any potential risk of intussusception induced by the vaccine (44). Nevertheless, comprehensive future investigations encompassing both the merits and risks of rotavirus vaccination are encouraged to lend further weight to these findings. We continue to champion the worldwide promotion of rotavirus vaccination, specifically in low-and middle-income nations where the infection is rife. Simultaneously, the differing risk/benefit scenarios for high-income and low-income countries cannot be ignored. In several high-income countries where rotavirus infections might be less common or less severe due to superior healthcare systems, the perceptible advantage of vaccination can be less prominent. This could explain why rotavirus vaccination is presently not endorsed in about half of the European nations. Irrespective of a country's economic standing, we recommend vigilant monitoring of clinical symptoms in infants and young children post-vaccination. If symptoms indicative of intussusception manifest, immediate diagnosis and intervention are imperative to curtail fatalities and disabilities due to intussusception (45, 46).

One potential reason we did not find a correlation between rotavirus vaccine administration and the incidence of intussusception is because we excluded studies involving the RRV-TV vaccine, which was withdrawn in 1999 due to safety concerns (15, 47). Heterogeneity in our analysis could be attributed to the varied designs and implementations of the included studies, variations in sample size, and event rates. It is worth highlighting that our study, primarily using a Randomized Controlled Trial (RCT) design, has a different methodology compared to those using a Self-Controlled Case Series (SCCS) design, like the study by Koch et al. which found a correlation between rotavirus vaccination and intussusception risk (48). The SCCS design is known for its effectiveness in controlling for fixed confounders, including those unmeasured or unknown, which makes it especially apt for evaluating vaccine safety. Research comparing SCCS to case-control designs has shown that the point estimates from both methods for several vaccines can be similar, but the precision of SCCS can lead to statistically significant effects due to its enhanced accuracy (49). This precision facilitated the identification of a small, but statistically significant increase in the risk for intussusception linked with rotavirus vaccination by Koch et al. (48). The choice of study design impacts the results, as our study using an RCT design produced different findings compared to those from SCCS-based studies. Our study, however, covers a broad spectrum by including all types of vaccines currently available and under clinical trials (14). While our findings align with many existing studies, we acknowledge the importance of diverse methodological approaches to a comprehensive understanding of rotavirus vaccination safety. Future research should continue to use a variety of study designs to provide a holistic and balanced perspective on this vital topic.

Our study also has some limitations that need to be improved in future research. Firstly, due to the limited number of included studies, we were unable to perform subgroup analysis on more factors, such as different age groups, genders, and races. Secondly, as a result of employing different data sources from various countries or regions in the included studies, there is a potential for information bias and omission bias (50). Thirdly, the quality of the included studies varies, with some studies having selection bias, confounding bias, measurement bias, etc., which may affect the credibility of the meta-analysis results (51). Fourth, the causal link between rotavirus vaccine administration and the onset of intussusception remains unclear. We cannot disregard the potential influence of other confounding or intervening factors. The findings of our meta-analysis demonstrate that these rotavirus vaccines do not significantly elevate the risk of intussusception. However, due to the heterogeneity of the research results and the fact that many vaccines were only investigated in single studies, our results need further research for confirmation. We suggest including more randomized controlled trials in the research to obtain more accurate and stable results.

## Conclusion

In summary, this study demonstrates that there is no significant correlation between the administration of the rotavirus vaccine and the occurrence of intussusception, regardless of the vaccine's brand or type. The advantages of administering the rotavirus vaccine significantly outweigh its risks, advocating its continued promotion. Future research should delve into the underlying biological mechanisms linking rotavirus vaccine administration with the onset of intussusception, while also considering the influence of varying factors such as age groups, gender, and ethnicities, in order to enhance the safety and efficacy of rotavirus vaccination.

## Data Availability

The original contributions presented in the study are included in the article/Supplementary Material, further inquiries can be directed to the corresponding authors.
